# Dynamics of postnatal upper airway bacteria colonization in preterm infants <1000g and bronchopulmonary dysplasia

**DOI:** 10.1038/s41598-025-29038-7

**Published:** 2025-11-28

**Authors:** Tina Frodermann, Ulrich Rochwalsky, Ariane Selting, Frank Oehmke, Katrin Gentil, Volkhard A. J. Kempf, Stephan Göttig, Michael Hogardt, Anastasia Anagnostou, Michael Schloter, Birte Staude, Jan Gertheiss, Harald Ehrhardt

**Affiliations:** 1 Department of General Pediatrics and Neonatology, Justus-Liebig-University, Feulgenstrasse 12, D-35392 Giessen, Germany; 2 Department of Paediatrics, Division of Neonatology, Goethe University Frankfurt, Frankfurt, Germany; 3 Department of Obstetrics and Gynecology, Justus-Liebig-University, Feulgenstrasse 12, D-35392 Giessen, Germany; 4 Institute of Medical Microbiology, Justus-Liebig-University, German Centre for Infection Research (DZIF), Schubertstrasse 81, D-35392 Giessen, Germany; 5 Institute for Medical Microbiology and Infection Control, University Hospital Frankfurt, Frankfurt, Germany; 6 Research Unit for Comparative Microbiome Analysis, Helmholtz Zentrum München GmbH, Ingolstädter Landstrasse 1, Neuherberg, Germany; 7 Department of Economics and Social Sciences, Statistics and Data Science Group, Helmut Schmidt University, Holstenhofweg 85, Hamburg, Germany; 8 Division of Neonatology and Paediatric Intensive Care Medicine, Department of Paediatrics and Adolescent Medicine, University Medical Centre Ulm, Eythstraße 24, D-89075 Ulm, Germany

**Keywords:** Preterm infant, Bronchopulmonary dysplasia, Risk, Bacterial colonization, Highly pathogenic bacteria, Facultative pathogenic bacteria, Upper airway, Lung, Diseases, Health care, Medical research, Microbiology, Risk factors

## Abstract

Aberrant microbial colonization of premature infants is increasingly recognized as a risk factor for severe acute morbidities. The aim of this study was to evaluate the correlation of bacterial upper airway colonization within the first 6 weeks of life in preterm infants <1000g and risk of moderate/severe bronchopulmonary dysplasia (BPD). In this retrospective two-center cohort study postnatal upper airway bacterial colonization of premature infants with a birth weight <1000g was analyzed. Bacteria were categorized into facultative- and highly pathogenic. Within 242 infants, a birth weight cutoff of 800g prevailed as the most relevant discriminator for risk of BPD. Furthermore, center, male sex, duration of antibiotic therapy, and delayed detection of facultative pathogenic bacteria after week 4 was associated with the development of BPD. Using classification tree analyses for the binary outcome, antibiotic therapy was more importance in infants <800g, whereas in those with a birth weight ≥800g, delayed colonization with facultative pathogenic bacteria was more relevant than antibiotic exposure. We add delayed colonization of the upper airway with facultative pathogenic bacteria to the risks for BPD. The variations of microbial colonization should be considered in future studies on the pathogenesis of BPD and new treatment modalities.

**Supplementary Information:**

The online version contains supplementary material available at 10.1038/s41598-025-29038-7.

## Introduction

Physiologic colonization of our body by microbiota from natural environment is a key driver of health mainly at early stages of life^1^. Preterm infants however are exposed to microbiota which are imprinted by the hospital and are often dominated by potentially harmful bacteria during their neonatal intensive care unit (NICU) stay^2–4^.

Bronchopulmonary dysplasia (BPD) is the chronic lung disease of preterm infants with lifelong limitations in lung function impacting physical capacity and quality of life^5–8^. The incidence of BPD remained unchanged during the last decades^9^. The lifesaving therapies oxygen supply and mechanical ventilation, as well as pre- and postnatal infections, constitute key BPD drivers^10^. In recent years, BPD research directions were expanded to address microbial dysbiosis of preterm infants at birth and during their NICU stay^11–13^.

Spontaneous delivery with exposure to microbes in the birth channel did not alter the pulmonary outcome compared to deliveries by caesarean section^14^. Microbiome studies in the amniotic fluid at preterm birth revealed specific microbiota signatures associated with the different severity stages of BPD^15^. Furthermore, postnatal colonization of the upper airway with highly pathogenic microorganisms was associated with moderate/severe BPD^16^. Longitudinal follow-up into adulthood showed a lifelong persisting microbial dysbiosis in infants with the diagnosis of BPD^17^. While all these studies cannot prove causality between specific microbiota structures and the evolution of BPD, preclinical studies in rodent models unraveled the pathomechanism of microbial lung injury^18–21^.

In addition, clinical routine management has a tremendous impact on the pulmonary outcome. While breast milk supply reduced the incidence of BPD, antibiotic exposure increased the risk which was traced back to changes in the microbiota^22,23^.

Here, we studied the dynamics of postnatal bacterial colonization in a two-center cohort of preterm infants <1000g^16,24^. Therefore, we performed a high throughput isolation of bacteria and used their taxonomy to define a risk matrix from facultative pathogenic to highly pathogenic bacteria^16^. Facultative pathogenicity is referring to bacteria that are not commonly associated with invasive infections in premature newborns, while bacteria with high pathogenicity are pathogens frequently causing invasive infections in this patient group as published before^16^. The focus of the analyses was set onto the association of BPD and bacterial upper airway colonization which was previously identified as the relevant site, and the first six weeks of life which covers the most vulnerable period for the evolution of BPD^16^. Most previous studies focused on the association of nosocomial infections and BPD. We had the hypothesis, that there will be differences in the dynamics of colonization of the upper airway with bacteria independent of causing nosocomial infections associated with BPD. While previous research has focused on highly pathogenic bacteria, to our knowledge, the dynamics of colonization with facultative pathogenic bacteria have not been evaluated before^16^. The results can guide research directions intended both to improve clinical care and the pulmonary outcome and risk prediction modelling.

## Methods

### Study design

**Fig. 1:**
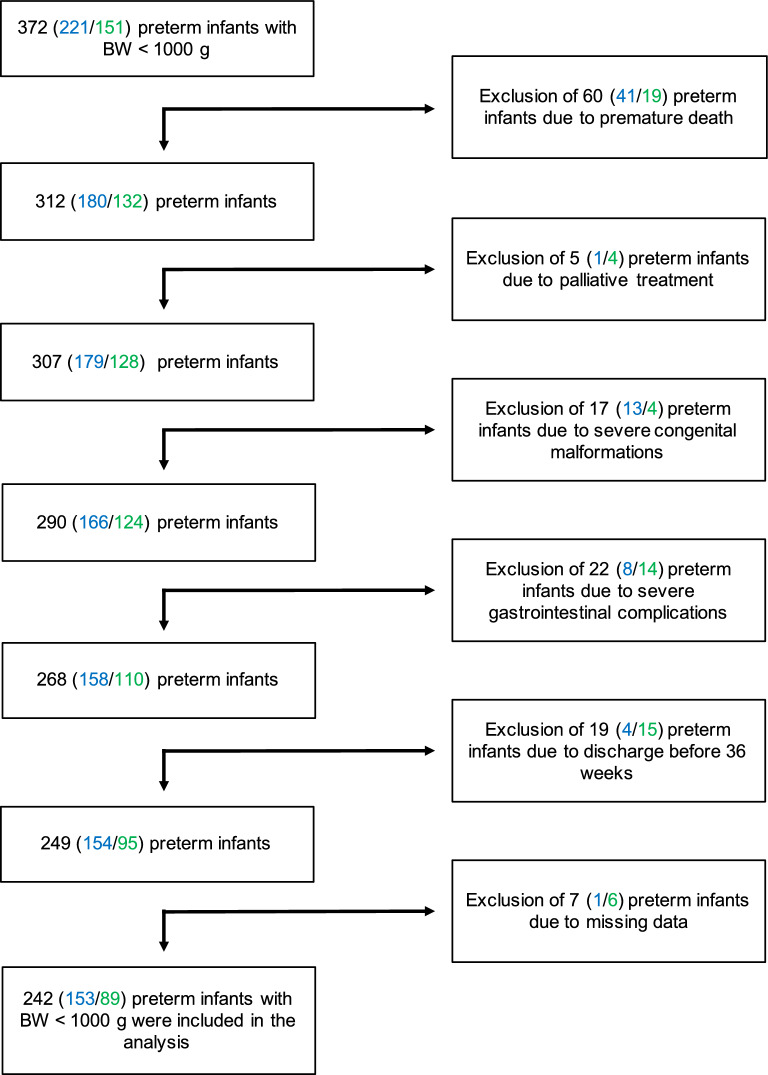
Flowchart of infants included into the analyses. Total number of infants available from both centers during the observation period, of infants included into the analyses and reasons for exclusion from the analyses are given as total number (Giessen site/Frankfurt site). BW, birth weight.

This retrospective two-center cohort study evaluated postnatal bacterial colonization during the first 6 weeks of life in preterm infants <1000g birth weight and <32 weeks´ gestation treated at the tertiary perinatal care centers Giessen (Justus Liebig University, Giessen, Germany) between January 2014 and December 2018 and Frankfurt (Goethe University, Frankfurt, Germany) between January 2017 and October 2021. We focused our analyses on describing associations and dynamic changes of upper airway colonization with facultative or highly pathogenic bacteria (detailed in Table 1) and BPD. From n= 372 eligible patients (n= 221 at the Giessen, n = 151 at the Frankfurt site), n= 96 preterm infants had to be excluded due to death before 36 weeks (n = 60), severe congenital malformations (n = 17) or transfer to another center before 36 weeks (n = 19). Deaths were excluded for lack of information on the BPD status and were mostly unrelated to pulmonary disease. Additionally, infants with focal intestinal perforation and necrotizing enterocolitis (n = 22) were excluded due to the high impact of prolonged ventilation periods and inflammatory episodes provoked by these events on the pulmonary outcome. Overall, n= 242 infants were available for the analyses, n = 153 from the Giessen and n = 89 from the Frankfurt site (Flowchart presented in Figure 1). The retrospective analysis was conducted according to the rules of the Declaration of Helsinki in its latest version of 2013 and was approved by the ethics committee of the Justus Liebig University Giessen (Az 97/14) and of the Goethe-University Frankfurt (2021/378). Due to the anonymous data documentation and the retrospective nature of the study, parental informed consent was waived.

**Table 1:** Table of bacteria with high and facultative pathogenicity detected in the upper airway by routine swabs during the first 6 weeks of life.

Bacteria with high pathogenicity	Upper airway (n = 242)
Enterococcus spp.	102 (42.15%)
Staphylococcus aureus	85 (35.12%)
Enterobacter spp.	61 (25.21%)
Klebsiella spp.	52 (21.49%)
Escherichia coli	45 (18.6%)
Proteus spp.	14 (5.79%)
Pseudomonas spp.	13 (5.37%)

Bacteria with facultative pathogenicity	
Coagulase negative *staphylococci*	228 (94.21%)
Alpha-hemolytic* streptococci*	55 (22.73%)

Displayed is the classification of bacteria as highly and facultative pathogenic with the number of occurrences in upper airway swabs. Only genera with a prevalence of >5% are shown. Following previously reported^16^ highly pathogenic species, *Escherichia coli*, *Serratia marcescens* (not shown), *Staphylococcus aureus* and *Stenotrophomonas maltophilia* (not shown) were counted on the species level. Coagulase negative staphylococci were grouped into bacteria with facultative pathogenicity. Streptococci were devided according to their hemolytic activity. An occurrence is counted if the species was found in upper airway swabs of a patient during the first 6 weeks of life, every genus/species is only counted once per patient.

### Longitudinal screening for bacterial upper airway colonization

Preterm infants underwent upper airway microbial examination of the nasal and oral cavity during routine clinical care at birth and weekly thereafter as recommended by the German Commission for Hospital Hygiene and Infectious Disease Prevention at the Robert Koch Institute (RKI) in 2013. Swabs (Microbiotech S.r.l., Maglie, Italy) were taken by qualified nursing staff and promptly transferred to the microbiological laboratory and analyzed by an cultivation based approach using blood, chocolate, and MacConkey agar plates (Thermo Fisher Scientific, Waltham, MA) at the Giessen site^16^. At the Frankfurt site, an agar-based swab (Transystem sterile transport swab, Hain lifescience, Nehren Germany) was used to collect patient samples. Culture was performed using Columbia blood, chocolate, MacConkey and Sabouraud-Agar plates (Thermo Fisher Scientific, Waltham, MA).

The categorization of bacterial strains into highly pathogenic bacterial species and those with facultative pathogenicity was executed based on the results of our previous study on the topic and on the published literature and the datasets from the German National Reference Centre for the Surveillance of Nosocomial Infections^16,25–28^. The following bacteria were considered as highly pathogenic bacteria covering twelve different genera and species (*Escherichia coli*, *Enterobacter* spp., *Enterococcus* spp., *Klebsiella* spp., *Pseudomonas* spp., *Serratia marcescens*, *Staphylococcus aureus*, *Citrobacter* spp., *Haemophilus* spp., *Proteus* spp., *Stenotrophomonas maltophilia*, Streptococcus group A and B) while all other bacteria were clustered as bacteria with facultative pathogenicity (Table 1). Due to their ubiquity and physiologic colonization in breastfed infants, coagulase-negative *staphylococci* were not classified as highly pathogenic despite their contribution to nosocomial infections^29,30^.

### Data acquisition and parameter definition

All patient metadata were extracted from the electronic patient management systems and the digitally archived paper file records. The datasets were entered into an SPSS (version 26.0.0.0, IBM, Armonk, NY) databank.

The following maternal and infant baseline parameters were recorded: gestational age (GA), birth weight (BW), sex, birth as singleton or multiple, premature rupture of membranes (PROM) >18 hours before delivery, reason for delivery separated into amniotic infection (AIS), preeclampsia/eclampsia/HELLP, intrauterine growth restriction (IUGR) and other causes, intraventricular hemorrhage (IVH) and retinopathy of prematurity. Antenatal steroid application (ANS) was categorized into no ANS, incomplete course (< 24 hours), completed course with delivery between 24 hours and 7 days after the first application, and ANS > 7 days before birth. Provision of an ANS booster before delivery was included in the completed course category. AIS was counted when at least one clinical criterion was fulfilled as published previously: histologic chorioamnionitis, amniotic fluid interleukin-6 > 3600 pg/ml, premature contractions during tocolytic therapy or PROM^15^. Small for gestational age (SGA) status was defined as BW, length, and head circumference below the 10^th^ percentile of the German perinatal registry percentiles^31,32^. Breast milk supply was documented independent of the quantity. Antibiotic exposure was documented separately for initiation directly after birth, any application, and the total duration of exposure within the first six weeks of life. First- and second-line antibiotic regimes differed between the two centers: in Giessen, the first-line therapy consisted of ampicillin plus gentamicin, while the second-line regimen included tazobactam plus vancomycin. In Frankfurt, first-line therapy consisted of ampicillin plus cefotaxime until January 2020, and ampicillin/sulbactam plus cefotaxime thereafter; the second-line regimen included ceftazidime plus vancomycin. For hygiene measures including prevention of nosocomial infection and lower airway colonization with bacteria, both centers applied the national recommendations by the Commission for Infection Prevention and Hygiene in Healthcare and Nursing (KRINKO). Late onset infections were counted by applying the criteria from the German NICU nosocomial infection surveillance system (NEO-KISS). Respiratory parameters comprised the total duration of invasive mechanical ventilation (IMV). BPD disease severity was categorized into no, mild, moderate, or severe BPD according to the most widely used 2001 NICHD consensus definition^33^. To account for alternative forms of respiratory support, continuous positive airway pressure (CPAP) during high-flow nasal cannula support and of the fraction of oxygen provided by low-flow nasal cannula were performed relying on the published conversions^31^.

### Statistical analysis

Patient baseline characteristics were compared using the Wilcoxon rank sum test, Chi-squared, and Fisher test, depending on the variables’ scale level and distribution. Statistical analysis was directed towards clustering disease severity stages for binary analyses into no/mild and moderate/severe BPD^34^. The first detection of bacterial colonization was categorized into weeks 1 to 6 after birth, and cases with no detection were grouped as >6. As the variability of characteristics was too low for each center, analyses were directed towards the total two-center cohort. For primary analysis, comparisons were made using logistic regression. Data analyses on bacterial colonization and risk of BPD are presented after risk adjustment for center, BW, sex, multiple births, ANS exposure, AIS, and duration of antibiotic therapy. GA and SGA status were not added to the adjustment model as both items are in collinearity with BW. The final adjustment model was chosen via AIC-based, backward selection with the known risk factors ANS, AIS, antibiotic therapy, and multiple births added manually. The effects of bacterial colonization were investigated using Analysis of Deviance/χ^2^-tests and Wald tests of contrasts. For reasons of interpretability, bacterial colonization was (re-)coded such that regression coefficients directly give contrasts of adjacent weeks^35^. As a confirmatory analysis, generalized additive (logistic) regression models were fit with smooth and (potentially) nonlinear effects of bacterial colonization, BW, and duration of antibiotic therapy^36,37^. To investigate potential interactions of risk factors and to lay the ground for potential follow-up studies through exploratory data analysis, a decision tree in terms of a “conditional inference tree” and a corresponding random forest were trained with default settings of the party package in R (minimum number of observations in a node = 20, minimum number of observations in a termina node = 7, threshold of 1 - p-value that must be exceeded to implement a split = 0.95, number of trees to grow in a forest = 500, number of input variables randomly sampled as candidates at each node = 5)^38,39^. Statistical analyses were executed with R version 4.3.1^40^. Significance was accepted with p<0.05.

## Results

Details on the 242 included preterm infants and reasons for exclusion are presented within the flowchart (Figure 1). Cohorts from both sites did not differ significantly in maternal and neonatal baseline characteristics (Table 2). Significant differences existed in the postnatal respiratory support and antibiotic therapy management while the rate of breast milk provision was similar (Table 2). 54% and 74% of infants required mechanical ventilation, and the mean duration of invasive mechanical ventilation was 1 and 4 days at the two centers, respectively (Table 2). Most infants fulfilled the BPD criterion without significant differences between the centers^31,33^. However, more moderate/severe BPD cases were diagnosed at the Giessen site (Table 2).

**Table 2:** Characteristics of preterm infants of the Giessen and Frankfurt center included in the analyses.

	Giessen	Frankfurt	**Total cohort**
	(n = 153)	(n = 89)	(n = 242)
Gestational age (days)	187 [179; 194]	186 [177;193]	186 [178;194]
Birth weight (g)	810 [690;930]	800 [620;950]	800 [680; 930]
SGA	12 (0.08)	11 (0.12)	23 (0.1)
NA	0 (0.0)	2 (0.02)	2 (0.01)
Male	73 (0.48)	43 (0.48)	116 (0.48)
Singleton	90 (0.59)	59 (0.66)	149 (0.62)
ANS^†^			
No	6 (0.04)	7 (0.08)	13 (0.05)
<24 h	18 (0.12)	10 (0.11)	28 (0.12)
24 h – 7 h	75 (0.49)	34 (0.38)	109 (0.45)
> 7 days	54 (0.35)	21 (0.24)	75 (0.31)
NA	0 (0.0)	17 (0.19)	17 (0.07)
PROM	37 (0.24)	24 (0.27)	61 (0.25)
Cause of delivery			
AIS	68 (0.44)	42 (0.47)	110 (0.45)
Preeclampsia/Eclampsia/HELLP	24 (0.16)	17 (0.19)	41 (0.17)
IUGR	20 (0.13)	6 (0.07)	26 (0.11)
Other	41 (0.27)	24 (0.27)	65 (0.27)
Intubation^**^	83 (0.54)	66 (0.74)	149 (0.62)
IMV (days)^†^	1 [0;4]	4 [0;13]	1 [0;8]
Breast milk supply	142 (0.93)	77 (0.87)	219 (0.9)
Antibiotic therapy started directly after birth^†^	113 (0.74)	83 (0.93)	196 (0.81)
Total duration antibiotic therapy (days)^†^	5 [5;10]	13 [7;19]	7 [5;14]
Nosocomial infection	23 (0.15)	13 (0.15)	36 (0.15)
BPD	128 (0.84)	74 (0.83)	202 (0.83)
NA	0 (0.0)	2 (0.02)	2 (0.01)
BPD severity**			
No/mild	103 (0.67)	72 (0.81)	175 (0.72)
Moderate/severe	50 (0.33)	15 (0.17)	65 (0.27)
NA	0 (0.0)	2 (0.02)	2 (0.01)
IVH			
No	131 (0.86)	71 (0.8)	202 (0.83)
Grade 1	5 (0.03)	4 (0.04)	9 (0.04)
Grade 2	4 (0.03)	5 (0.06)	9 (0.04)
Grade 3	1 (0.01)	2 (0.02)	3 (0.01)
IPH	12 (0.08)	7 (0.08)	19 (0.08)
NA	0 (0.0)	1 (0.01)	1 (0.0)
ROP^†^			
No	56 (0.37)	59 (0.66)	115 (0.48)
Grade 1	37 (0.24)	7 (0.08)	44 (0.18)
Grade 2	30 (0.2)	16 (0.18)	46 (0.19)
Grade 3	29 (0.19)	2 (0.02)	31 (0.13)
Grade 4	0 (0.0)	1 (0.01)	1 (0.01)
Grade 5	0 (0.0)	0 (0.0)	0 (0.0)
NA	1 (0.01)	4 (0.04)	5 (0.02)

Maternal and neonatal characteristics are presented separately for the Giessen and Frankfurt center and the total cohort. Qualitative data is presented as n with proportion in brackets. Quantitative data is presented as median with 1^st^ and 3^rd^ quartile in square brackets. Missing data has been marked as “NA”. For statistical analyses, Wilcoxon, Chi-squared, and Fisher tests were used as appropriate; p-values were calculated in R 4.3.1^41^, comparing characteristics of the Giessen and Frankfurt sites. SGA, small for gestation age; ANS, antenatal steroids; PROM, premature rupture of membranes; AIS, amnion infection syndrome; IUGR, intrauterine growth restriction; IMV, invasive mechanical ventilation; BPD, bronchopulmonary dysplasia; IVH, intraventricular hemorrhage; IPH, intraparenchymal hemorrhage; ROP, retinopathy of prematurity. P-value annotation: * < 0.05, ** < 0.01, ^†^ < 0.001.

We directed the analyses towards clustering disease severity stages for the binary analysis of no/mild and moderate/severe BPD^16,31,34^.

### Bacterial colonization of the upper airway within the first six weeks of life

**Fig. 2:**
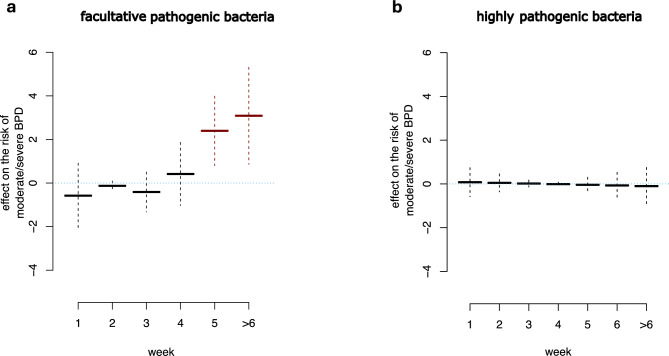
Effect size of timing of bacterial colonization of the upper airway on the severity of bronchopulmonary dysplasia Effect sizes on the logit scale of bacterial colonization of the upper airway with bacterial species with facultative (**a**) and high (**b**) pathogenicity were studied in a generalized (logit) additive model with response moderate/severe (1) vs. no/mild (0) bronchopulmonary dysplasia (BPD). Depicted is the effect size (together with approximate, pointwise 95% confidence intervals) of the first colonization of the upper airway separated by postnatal weeks for the development of moderate/severe BPD. Positive values of the effect size on moderate/severe BPD indicate an increased risk while negative effect sizes indicate risk reduction. No detection of bacteria within the first 6 weeks of life was labelled as >6. When no first detection of bacteria was documented in one week, this week is not displayed. Analyses were executed using R 4.3.1^40^ and add-on packages mgcv 1.9-0.9^36^ and ordPens 1.0.0^55^. Significant effects are emphasized in red.

**Fig. 3:**
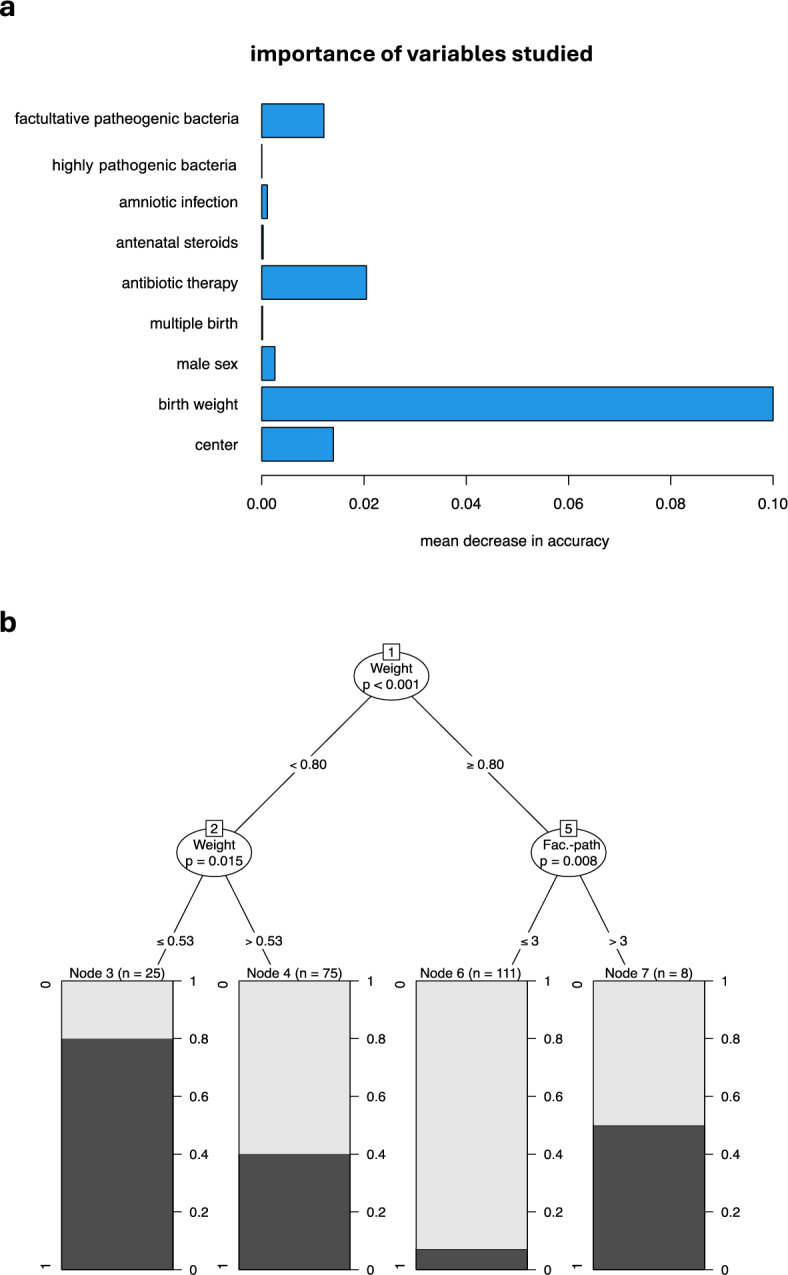
Relative importance of variables studied and decision tree algorithm for items most relevant for BPD (**a**) Depicted is the relative importance of each variable studied for the pulmonary outcome of moderate/severe BPD using a random forest with the binary outcome moderate/severe vs. no/mild BPD. Analyses were executed using R 4.3.1 and add-on package party 1.3–13.3^38,39^. (**b**) Decision tree analysis of items most relevant for the outcome moderate/severe vs. no/mild bronchopulmonary dysplasia using conditional inference trees was directed towards the dichotomous separation of the pulmonary outcome into no/mild and moderate/severe BPD. Analyses were done with R 4.3.1 and party 1.3–13.3^38,39^. Low birth weight <800g was the most significant risk factor, followed by a a further cut-off at weight ≤530g within the <800g group, and colonization with facultative pathogenic (Fac.-path.) bacteria after week 3 in the group ≥800g. The number of patients in the final subgroups (nodes) is indicated by “n = “, and the prevalence of moderate to severe BPD (1, dark grey) vs no or mild BPD (0, light grey) in each node is indicated by the bars at the bottom level of the tree.

We first analyzed the colonization with highly pathogenic bacterial-species in the Giessen cohort. As published before, early colonization in the first or second week of life showed associations with moderate/severe BPD. However, statistical analyses no longer detected significant differences when the weekly parametric approach was applied, and each week was considered separately. For the week 2 to 3 comparison, the estimate would still be significant on the 10% level (p-value = 0.0836, Table S1)^16,37^. However, when we added the infants treated at the Frankfurt site, the association of colonization with bacteria of high pathogenicity and BPD was much weaker, arguing for a center-specific effect. To explore general associations within our primary analysis, we pooled data from both Giessen and Frankfurt for all further analyses (Table 3). The variables center, lower BW, and male sex showed a statistically significant effect. Also, the duration of (any) antibiotic therapy seems to affect the risk of BPD, although not significantly on the 5% level. Thanks to the increased sample size of the two-center study, the information available on facultative pathogenic bacterial species could be exploited together with the highly pathogenic bacteria. The estimate (5.09, p-value = 0.0057) prevailed in favor of colonization with facultative pathogenic bacterial species before the 5^th^ week of life (Table 3). When we changed the statistical model from the generalized linear to the generalized additive model to evaluate the robustness of our results, incorporating smooth terms of bacterial colonization, consistent results prevailed for the colonization pattern with facultative pathogenic bacteria. Specifically, retarded colonization beyond week 4 of life increased the risk of developing BPD substantially and significantly (Table 4 and Figure 2A+B). Next, we considered potential interactions of risk factors by using a random forest and evaluated the importance of the variables studied for the BPD outcome. BW had by far the largest impact on the risk of BPD (Figure 3A). Any antibiotic therapy and the colonization with bacteria with facultative pathogenicity together with the center were the variables with the most considerable impact after BW, even before sex (Figure 3A).

**Table 3:** Logit model of the risks for moderate/severe BPD for the total cohort.

	Estimate	Standard error	z-value
(intercept)**	4.949	1.70736	2.899
Frankfurt site^†^	−2.11368	0.62782	−3.367
Birth weight (kg)^†^	−8.95953	1.65432	−5.416
Sex (male)*	1.11412	0.46151	2.414
Multiple births	−0.50356	0.47915	−1.051
ANS (vs. No)			
< 24 h	−0.15105	1.19822	−0.126
24 h – 7 days	0.03627	1.00548	0.036
> 7 days	−0.1395	1.08092	−0.129
AIS	0.56031	0.49578	1.13
Duration of antibiotic therapy	0.05799	0.03292	1.762
**Upper airway colonization with highly pathogenic bacteria**			
Week 1/2	−1.47856	1.0539	−1.403
Week 2/3	−0.05833	0.69657	−0.084
Week 3/4	0.46726	0.72479	0.645
Week 4/5	−0.3111	0.78706	−0.395
Week 5/6	0.24563	0.85432	0.288
Week 6/>6	−0.29472	0.97304	−0.303
**Upper airway colonization with facultative pathogenic bacteria**			
Week 1/2	1.29637	1.00284	1.293
Week 2/3	−0.54765	0.71094	−0.77
Week 3/4	−0.92393	1.68691	−0.548
Week 4/>4**	5.09094	1.84085	2.766

AIC-based, backward selection was employed for all infants from both centers. For bacteria with facultative pathogenicity, weeks >4 were collapsed due to the small number of children in the respective upper categories. P-value annotation: * < 0.05, ** < 0.01, ^†^ < 0.001.

**Table 4:** Generalized (logit) additive model of the risk for moderate/severe BPD for the total cohort.

***Parametric coefficients***			
	Estimate	Std. error	z-value
(intercept)**	4.76187	1.50746	3.159
Frankfurt site^†^	−2.20224	0.58756	−3.748
Birth weight (kg)^†^	−8.38939	1.57198	−5.337
Sex (male)**	1.16259	0.43747	2.658
Multiple births	−0.46716	0.44729	−1.044
ANS (vs. No)			
< 24 h	−0.50057	1.09174	−0.459
24 h – 7 days	−0.12842	0.93936	−0.137
> 7 days	−0.2131	0.98063	−0.217
AIS	0.52586	0.44782	1.174
Duration of antibiotic therapy	0.05621	0.02879	1.952

***Approximate significance of smooth terms***			
	**edf**	**Ref.df**	**Chi.sq**
Upper airway colonization with highly pathogenic bacteria	1	1	0.054
Upper airway colonization with facultative pathogenic bacteria*	3.072	3.762	12.358

Generalized additive (logistic) regression model was used analogously to Table 3 but with smooth effects of upper airway colonization with highly pathogenic bacteria and bacteria with facultative pathogenicity. Analyses were executed using R 4.3.1^41^ and add-on packages mgcv 1.9-0.9^37^ and ordPens 1.0.0^56^. The analyses revealed significance of smooth terms for the timepoint of colonization with facultative pathogenic bacteria. Fitted smooth effects are shown in Figure 2. P-value notation: * < 0.05, ** < 0.01, ^†^ < 0.001.

### Patient characteristics and pulmonary outcome using decision tree analyses

**Fig. 4:**
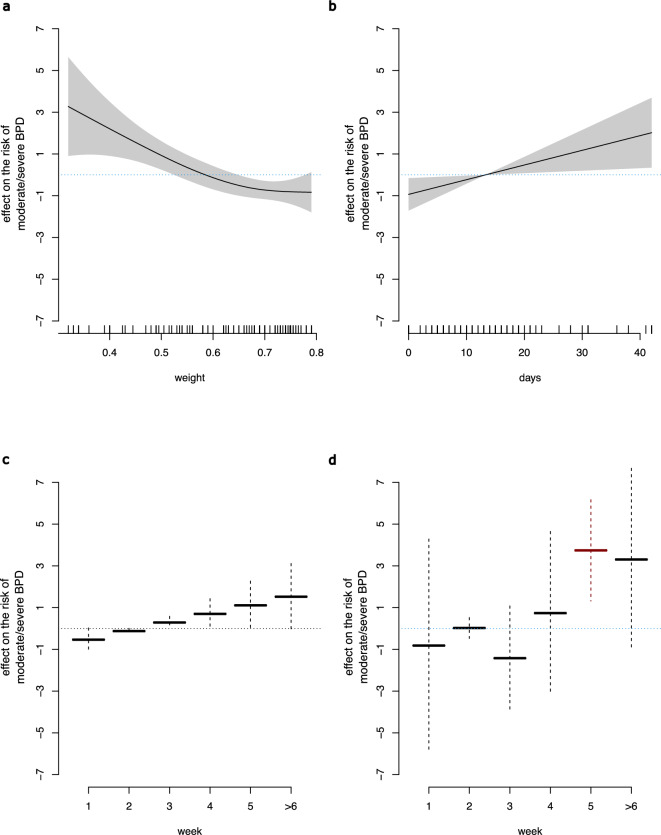
Relative contribution (effect sizes on the logit scale) of birth weight, duration of antibiotic therapy and upper airway colonization with facultative pathogenic bacterial species in a generalized (logit) additive model with outcome moderate/severe vs. no/mild BPD segregated by birth weight Depicted is the impact (smooth, potentially non-linear effects, together with pointwise, approximate 95% confidence intervals) of birth weight (**a**), and duration of antibiotic therapy (**b**) in the birth weight strata <800g as well as the impact of upper airway colonization with facultative pathogenic bacteria in the birth weight strata <800g (**c**). Furthermore, the effect sizes of upper airway colonization with facultative pathogenic bacteria are displayed for infants with a birth weight ≥800g (**d**). Analyses were done analogously to Figure 2. Positive values of the effect size on moderate/severe BPD indicate an increased risk while negative effect sizes indicate risk reduction. Significant effects are emphasized in red color.

Finally, we directed our analysis strategy to decision tree analysis, including the clinical risk factors and germ classification. This approach allowed hierarchical ordering and evaluating potential interactions between the items studied for dichotomous BPD severity classification. Again, BW was the most relevant variable with a cutoff of <800g (Figure 3B). Second-level segregation prevailed a BW ≤530g as a further increased risk for moderate/severe BPD in infants with a BW <800g, while the retarded colonization with facultative pathogenic bacterial species was of highest relevance in infants with a BW ≥800g (Figure 3B). Separate consideration of the two BW strata in the generalized additive model and evaluating center, sex, antibiotic therapy, and colonization with facultative pathogenic bacteria showed that antibiotic therapy is highly relevant (besides BW and center) in infants with a BW <800g (Figure 4A+B, Figure 4C, Table S2). However, in the strata with a BW ≥800g, the first colonization of the upper airway with facultative pathogenic bacteria is among the most important predictors (together with center and sex, see Figure 4D). By contrast, further segregation of birth weight and antibiotic therapy did not significantly affect the risk of developing BPD in the BW ≥800g strata (Table S3).

## Discussion

Our data add an additional dimension to the association studies of microbial colonization and development of BPD. Our data provide evidence that the timepoint of the first colonization of the upper airway with facultative pathogenic bacterial species is of higher relevance for the pulmonary outcome than the colonization with bacteria with high pathogenicity. Whereas previous studies primarily focused on the association of nosocomial infections and BPD, our study indicates an independent association between colonization pattern and BPD, even after adjusting for the duration of antibiotic therapy as a proxy of infection^10,19,23^. Further, we describe center differences in the association of early detection of bacteria with high pathogenicity in the upper airway and BPD. These data are important to guide readouts of improvement strategies in clinical care. Lastly, the clinical items BW and antibiotic therapy, together with the dynamics of upper airway colonization with facultative pathogenic bacterial species are suited to improve the prediction accuracy and deserve consideration within efforts to improve prediction models for BPD.

The microbiome in the neonate is actively shaped during the first weeks of life and the presence of bacteria with facultative pathogenicity is known to protect the newborn from early inflammation and later development of inflammatory diseases like asthma^41–43^. Our data on the dynamics of colonization with facultative pathogenic bacterial species are in line with results from previous research studies where early exposure of the immune system to low inflammatory stimuli induced immunotolerance and attenuated reactivity to a second overt stimulus^44,45^. It is therefore necessary to respect the timing of airway colonization^11,12,16^.

Overall, our data add further evidence that the dynamics of microbial colonization are highly relevant for the BPD outcome and that the multiple contributors to BPD must be held accountable for the still unmet need to identify one common potent therapeutic strategy that addresses the total spectrum of BPD contributors^46^.

### Strengths and limitations

The major strength of our study is the analysis of a two-center cohort with large variations in clinical routine practices, including respiratory management and antibiotic regimes, that are important confounders to the topic. Additionally, clinical routine management relevant to the outcome was not basically changed during the observation period, infants were treated according to current guidelines and recommendations including high rate of breast milk provision, and infants with severe comorbidities relevant to the BPD outcome were excluded from the analyses. The relevance of our study approach is secured by the fact that in preterm infants on invasive mechanical ventilation bacterial signatures detected in the upper airway are retrieved in their lungs, arguing for shaping of the lower respiratory tract bacterial milieu by the upper airway microbiome^47^. We used state-of-the-art statistical analyses, including risk adjustment for confounders. Cluster analyses using decision trees highlight the high relevance of aberrant dynamics in microbial colonization and antibiotic therapy for the BPD outcome besides BW.

However, we acknowledge several limitations in our study. First, we were not able to break down the results to the single bacterial species level despite the large number of patients included in the analyses as infants get irregularly colonized with bacteria with high pathogenicity, and some bacteria are rarely detected^27–30^. We were not able to control for potential site-specific effects of variations in swab types used and transport conditions. We observed an association of postnatal antibiotic exposure and BPD, consistent with previous findings. However, it was not possible to specify alterations in microbial colonization by antibiotics due to their frequent use in our study population. Further segregation of center-specific variations in start, duration, and selected regimes of antibiotic therapy was not possible as these data are not available within our datasets in such granularity^23^. Although first- and second-line antibiotic regimes differed between the two centers, the association between antibiotic exposure and BPD risk remained evident, even though the center with higher antibiotic exposure (Frankfurt site) had a lower unadjusted BPD prevalence. This and the additional adjustment for center argues against confounding by a center effect for this risk factor. More complex modeling of center-specific effects was not feasible due to the number of centers (n=2). A subgroup analysis was conducted for the Giessen center, but it was limited in power due to the small sample size, and was not conducted for the Frankfurt center due to the lower number of patients. The distribution of bacterial species was diverse and in line with the published literature, with a dominance of bacteria like coagulase-negative staphylococci, *Escherichia, Enterobacter,* and *Klebsiella* species^25–28^. When putting the bacteria into clusters, the variability was high enough to describe statistically significant differences with p-values <0.01, underlining the relevance of the data. Nowadays, high-throughput analysis by molecular barcoding is becoming more and more established within research projects and allows more precise detection of bacterial DNA diversity in niches that harbor a diverse microbiome. However, it does not allow for the identification of viable bacteria, and the interpretation of the data is often difficult. Furthermore, the extensive use of devices and measures in the NICU, including nasogastric tubes, CPAP devices, and regular suctioning of the upper airway to prevent secret retention, constitutes a risk for RNA contamination. By using routine bacterial culture techniques, we were able to detect a viable bacterial spectrum in the upper airway on a weekly basis and dynamic changes in their presence.

## Conclusions

Our data add new knowledge on the relevance of bacterial colonization after birth for the pulmonary outcome in preterm infants. While the focus was set on highly pathogenic bacteria and nosocomial infections before, the results presented here open a further dimension putting the focus on facultative pathogenic bacterial species and the dynamic changes over time. Our data do not allow a conclusion whether facultative pathogenic bacteria act as true “protective” commensals, or whether their delayed appearance is a surrogate marker of broader dysbiosis, e.g., caused by differences in respiratory and non-respiratory management. It will be essential to integrate analyses on bacterial colonization with further investigations, such as assessments of the inflammatory status from tracheal aspirates, longitudinal X-ray changes, infectious laboratory markers and the selection and escalation of antibiotic therapy and respiratory support.

The tight association between bacterial colonization and BPD reflects its high ranking within the multifactorial origins of BPD and demands research priority directed to modulate the bacterial structures in the airways towards a physiologic milieu. When taking all the previously published data on the topic into consideration, it is not surprising that measures intended to preserve the physiologic microbiome, particularly the provision of breast milk and care bundles to reduce antibiotic exposure to a minimum, are able to reduce the BPD burden^48–50^. Unfortunately, they are of limited efficacy, and further research is needed to better understand the microbial axes in preterm infants. The results of our studies on prenatal and postnatal microbial colonization demand further studies on this topic^3,15,16^. Current evidence highlights the focus on therapeutic strategies that support the early colonization of the upper airway with facultative pathogenic bacterial species. As probiotic therapy has failed to prove any benefit for BPD, alternative modes of application or novel formulations are urgently needed that respect strain-specific effects or combine probiotics with other classes of microbiota shaping strategies^42,50–54^. Clinicians should be encouraged to further optimize the provision of breast milk feeding and to implement bundles to further reduce the antibiotic exposure until novel strategies will be available.

Our concept of aberrant bacterial structure dynamics in the evolution of BPD should be exploited on a mechanistic level. It can be relevant to all further lung diseases where aberrant immune activation takes center stage in disease origin and progression.

## Supplementary Information

Supplementary Information.

## Data Availability

Deidentified datasets, including data dictionaries, will be made available to researchers who provide a reasonable and methodologically sound research proposal that is congruent with the goals of the ethics approval and that does not compete with further scheduled analyses of the authors. Proposals should be submitted to the corresponding author.
