# Supplementary material for: Dynamics of postnatal upper airway bacteria colonization in preterm infants <1000g and bronchopulmonary dysplasia
Source: Sci Rep. 2025 Nov 28;15:42558. doi: 10.1038/s41598-025-29038-7 (PMC12663399; doi:10.1038/s41598-025-29038-7)
Supplement: Supplementary file 1 — Supplementary Information. [file 41598_2025_29038_MOESM1_ESM.docx]

**Supplement: Dynamics of postnatal upper airway bacteria colonization in preterm infants <1000g and bronchopulmonary dysplasia**

**Table S1.** Confounder (logit) model of the risks for moderate/severe BPD for the Giessen center

**Table S2.** Generalized (logit) additive model of the risks for moderate/severe BPD for infants with a birth weight < 800g

**Table S3.** Generalized (logit) additive model of the risks for moderate/severe BPD for infants with a birth weight ≥ 800g

**Table S1. Confounder (logit) model of the risks for moderate/severe BPD for the Giessen center**

|  | **Estimate** | **Standard error** | **z-value** |
| --- | --- | --- | --- |
| **(intercept)**** | 5.57149 | 1.91559 | 2.908 |
| **Birth weight (kg) ^†^** | -7.13323 | 1.75996 | -4.053 |
| **Sex (male)*** | 1.13160 | 0.47412 | 2.387 |
| **Multiple births** | 0.12945 | 0.49335 | 0.262 |
| **AIS** | -0.45862 | 0.50450 | -0.909 |
| **ANS (vs. No)** |  |  |  |
| **< 24h** | -0.13353 | 1.46288 | -0.091 |
| **24 h – 7 days** | 0.36858 | 1.26344 | 0.292 |
| **> 7 days** | -0.19409 | 1.34207 | -0.145 |
| **Duration of Antibiotic therapy** | 0.06822 | 0.03959 | 1.723 |
| **Upper airway colonization with highly pathogenic bacteria** | | | |
| **Week 1/2** | -1.85603 | 1.31337 | -1.413 |
| **Week 2/3** | -1.25857 | 0.72737 | -1.730 |
| **Week 3/4** | 0.94484 | 0.76350 | 1.238 |
| **Week 4/5** | 0.10137 | 0.85418 | 0.119 |
| **Week 5/6** | 0.31867 | 0.92358 | 0.345 |
| **Week 6/>6** | 0.48233 | 1.03337 | 0.467 |

The severity of bronchopulmonary dysplasia (BPD) was separated into no/mild BPD (0) and moderate/severe BPD (1) as the binary response. Covariates in the logit model were birth weight, sex (1: male, 0: female), multiple births (yes/no), amniotic infection syndrome (AIS), antenatal steroids (ANS), and duration (days) of antibiotic therapy during the first six weeks of life. AIC-based, backward selection was run on the Giessen cohort, but (potential) risk factors multiple births, ANS, AIS, and upper airway colonization with highly pathogenic bacteria were added manually. Week x/y gives the contrast between weeks x and y. P-value annotation: * < 0.05, ** < 0.01, ^†^ < 0.001.

**Table S2. Generalized (logit) additive model of the risks for moderate/severe BPD for infants with a birth weight <800g**

| *Parametric coefficients* | | | |
| --- | --- | --- | --- |
|  | **Estimate** | **Standard error** | **z-Value** |
| (intercept) | 0.4874 | 0.3605 | 1.352 |
| Frankfurt site^†^ | -2.1491 | 0.6352 | -3.383 |
| Sex (male) | 0.5091 | 0.4644 | 1,096 |
| *Approximate significance of smooth terms* | | | |
|  | **edf** | **Ref.df** | **Chi.sq** |
| Birth weight** | 1.975 | 2.501 | 13.097 |
| Duration of antibiotic therapy* | 1.000 | 1.000 | 5.801 |
| Upper airway colonization with bacteria with facultative pathogenicity | 1.000 | 1.000 | 3.647 |

The model from Table 3 was restricted to infants with a birth weight <800g and risk factors center and sex. (Additional) smooth terms were included for birth weight, duration of antibiotic therapy, and upper airway colonization with bacteria with facultative pathogenicity. P-value annotation: * < 0.05, ** < 0.01, ^†^ < 0.001. Results show significance of smooth terms for birth weight and duration of antibiotic therapy but not for timepoint of upper airway colonization with facultatively-pathogenic bacteria. Fitted smooth effects are detailed in Figure 4a – c.

**Table S3. Generalized (logit) additive model of the risks for moderate/severe BPD for infants with a birth weight ≥800g**

| *Parametric coefficients* | | | |
| --- | --- | --- | --- |
|  | **Estimate** | **Standard error** | **z-Value** |
| (intercept) ^†^ | -3.3547 | 0.8469 | -3.961 |
| Frankfurt site* | -2.7271 | 1.3491 | -2.021 |
| Sex (male)* | 1.9299 | 0.9220 | 2.093 |
| *Approximate significance of smooth terms* | |  |  |
|  | **edf** | **Ref.df** | **Chi.sq** |
| Birth weight | 1.000 | 1.000 | 1.862 |
| Duration of antibiotic therapy | 1.000 | 1.000 | 0.902 |
| Upper airway colonization with bacteria with facultative pathogenicity* | 3.381 | 4.053 | 10.837 |

The model from Table 3 was restricted to infants with a birth weight ≥800g and risk factors center and sex. (Additional) smooth terms were included for birth weight, duration of antibiotic therapy, and upper airway colonization with bacteria with facultative pathogenicity. P-value annotation: * < 0.05, ** < 0.01, ^†^ < 0.001. Results show significance of smooth terms for timepoint of upper airway colonization with bacteria with facultative pathogenicity (fitted smooth effects are detailed in Figure 4d) but not for birth weight and duration of antibiotic therapy for this subgroup.
